# Task-irrelevant valence-preferred colors boost visual search for a singleton-shape target

**DOI:** 10.1007/s00426-023-01880-2

**Published:** 2023-10-11

**Authors:** Miloš Stanković, Hermann J. Müller, Zhuanghua Shi

**Affiliations:** 1 General and Experimental Psychology, Department of Psychology, Ludwig-Maximilians-Universität München, Munich, Germany; 2 Department of Psychiatry and Psychotherapy, University of Regensburg, Regensburg, Germany

## Abstract

Some studies have suggested that emotion-associated features might influence attentional capture. However, demonstrating valence-dependent distractor interference has proven challenging, possibly due to the neglect of individuals’ color–valence preferences in standard, averaged reaction-time (RT) measures. To address this, we investigated valence-driven attentional-capture using an association phase in which emotionally neutral vs. positive-feedback photographs were paired with two alternative target colors, red vs. green. This was followed by a test phase requiring participants to search for a pop-out shape target in the presence or absence of an emotion-associated color. In Experiments 1 and 2, this color could only appear in a distractor, while in Experiment 3, it appeared in the target. Analyzing the standard, averaged RT measures, we found no significant valence association or valence-modulated attentional capture. However, correlational analyses revealed a positive relationship between individual participants’ color–valence preference during the association phase and their valence-based effect during the test phase. Moreover, most individuals favored red over green in the association phase, leading to marked color-related asymmetries in the average measures. Crucially, the presence of the valence-preferred color anywhere in the test display *facilitated* RTs. This effect persisted even when the color appeared in one of the distractors (Experiments 1 and 2), at variance with this distractor capturing attention. These findings suggest that task-irrelevant valence-preferred color signals were registered pre-attentively and boosted performance, likely by raising the general (non-spatial) alertness level. However, these signals were likely kept out of attentional-priority computation to prevent inadvertent attentional capture.

## Introduction

Human behavior—and attention—are strongly driven by reward to ensure survival and well-being. People usually direct their attention to salient and behaviorally relevant stimuli, while ignoring non-salient and irrelevant distractors. Attention plays a central role in perception, cognition, and action, by prioritizing task-relevant over irrelevant sensory information to achieve behavioral goals. However, a non-salient object may be task-relevant, placing salience and relevance into conflict. Classical theories of attention assume two basic modes of attentional selection: stimulus- and goal-driven attention (e.g., Desimone & Duncan, 1995; Müller & Rabbitt, 1989; Theeuwes, 2019; Wolfe, 2021). Goal-driven attention is considered voluntary, involving a top–down set for task-relevant information; by contrast, stimulus-driven attention is considered involuntary, such as when attention is captured (bottom–up) by physically salient stimuli that may be irrelevant to the task at hand (i.e., outside the task set). Both modes of attention play an important role in individuals’ adaptation to the environment, by allocating attention based on behavioral goals (the task at hand) while remaining open to new stimuli (outside the task set) which may be survival-relevant. Thus, for example, the attentional set for task-relevant stimulus features may be breached by irrelevant but salient distractors that signal a potential reward.

Laboratory-based studies frequently use associative learning to connect rewards with arbitrary target features (Anderson & Halpern, 2017; Anderson et al., 2011; Bourgeois et al., 2016; Shi et al., 2020; Vuilleumier, 2015). For instance, during the initial association phase in the ‘value-driven attentional-capture paradigm’ (e.g., Anderson et al., 2011), high or low monetary rewards are associated with arbitrary stimulus features, such as the colors red or green (Anderson et al., 2011) or locations (Schlagbauer et al., 2014). These features are task-relevant in the initial phase, but become irrelevant distractors in the test phase. Typically, a high-reward distractor interferes more with search performance than a low-reward one, even though both types of distractor are of similar physical salience (Anderson, 2013; Anderson et al., 2011; but see Sha & Jiang, 2016). This pattern is suggestive of attentional capture by value-associated distractors, even in the absence of reward feedback (during the test phase).

**Fig. 1:**
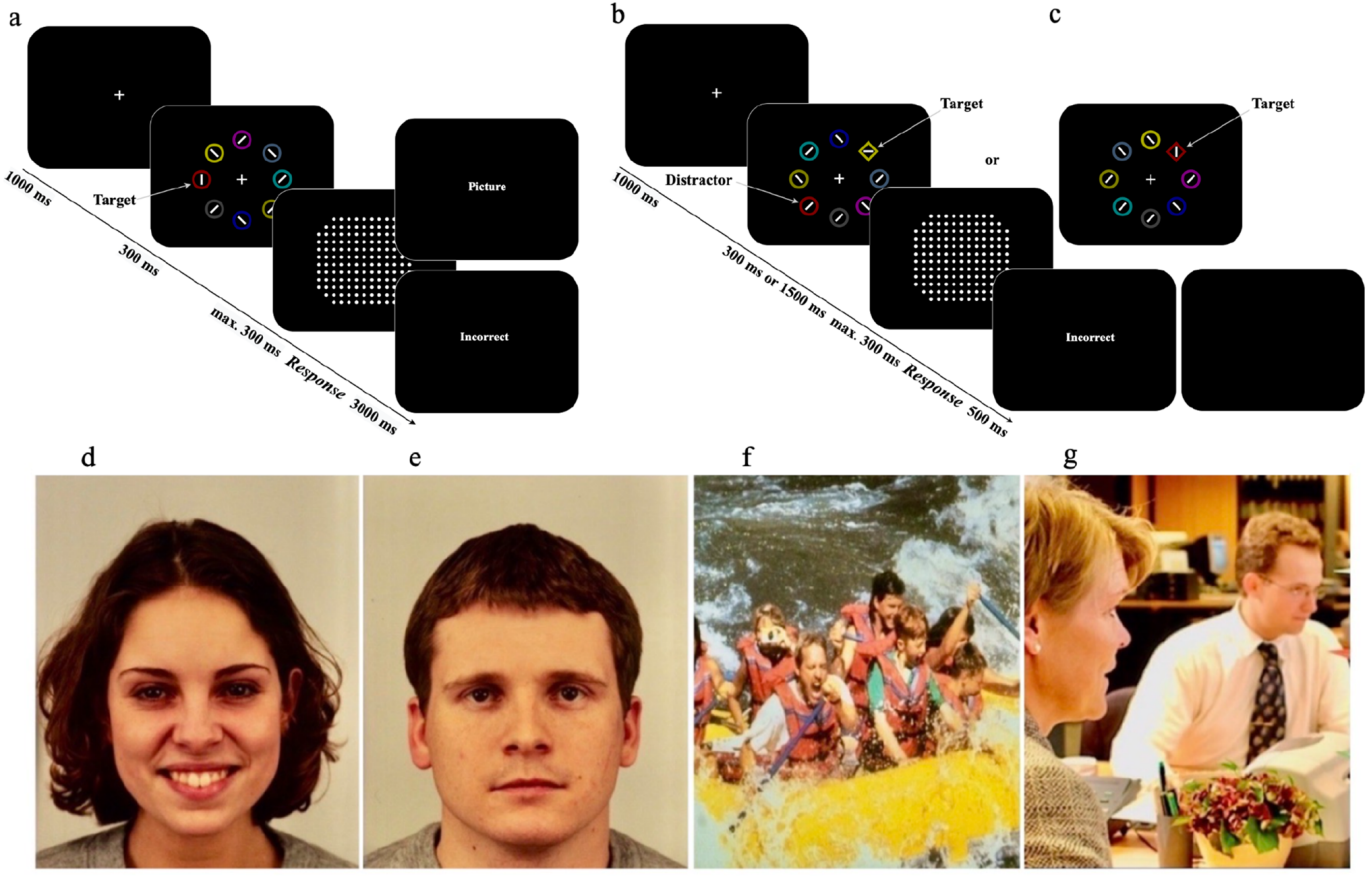
The emotion-driven attentional-capture paradigm used in Experiments 1, 2, and 3. **a** In the association phase, participants were instructed to find a (single) red or green ‘target’ circle (amongst heterogeneously colored non-target circles) and report whether the bar inside the target circle was oriented vertically or horizontally. Upon a correct response, participants were ‘rewarded’ by the presentation of a pleasant (**d** and, respectively, **f**) or neutral (**e** and, respectively, **g**) photograph; an incorrect response was followed by the text message “Incorrect” and the absence of a photograph. **b** In the test phase (Experiments 1 and 2), participants were instructed to report the orientation of the bar inside the singleton diamond (i.e., shape) ‘target’ amongst homogeneous non-target circles, disregarding the colors of the stimuli. **c** In the test phase of Experiment 3, instead of the reward-associated colors (red or green) appearing in a non-target circle (acting as ‘distractors’) as in Experiments 1 and 2, the singleton-diamond ‘target’ was red or green in two-thirds of the trials; in the remaining third, none of the (target or non-target) stimuli was ever red or green. The instruction was again to ignore the item colors, as the target could be reliably detected (on all trials) only by its odd-one-out shape. Examples of **d** a pleasant-valence face (AF20HAS - happy face); **e** a neutral-valence face (AM31NES - neutral face); **f** a pleasant-valence and high-arousal scene (IAPS_8370 - Rafting); and **g** a neutral-valence and low-arousal scene (EmoPics_124 - Office), from the Karolinska Directed Emotional Faces, International Affective Picture System, and EmoPics sets

Emotionally salient stimuli can also disrupt attention, similar to physically salient or reward-associated stimuli (Arnell et al., 2007). Emotionally significant stimuli typically attract attention more than neutral ones (Vuilleumier & Huang, 2009). Even neutral stimuli, when coupled with emotionally charged social feedback (e.g., an angry or happy face), can interfere with goal-oriented attention allocation (Anderson, 2016, 2017; Anderson & Kim, 2018; Kim & Anderson, 2020). For example, Anderson (2017) and Kim and Anderson (2020) probed valence-dependent attentional-capture effects in an experiment in which task-irrelevant color ‘distractors’ in the test phase were previously associated with emotional feedback stimuli (such as a happy or neutral face) in the preceding training phase (for an illustration of the paradigm as implemented in the present study, see Fig. 1). They hypothesized that distractor stimuli associated with high-valence feedback would command greater attentional capture than those associated with low-valence feedback. However, the presence of any distractor color, regardless of its valence association, was found to induce a similar performance cost relative to the baseline, when no distractor was present. This led to questions about whether the interference from distractors irrespective of their valence association reflected a search-history effect (i.e., any color searched for in the training ‘history’ were later on attentionally prioritized), rather than an emotion-history effect (i.e., specific colors searched for during training were associated with their correspondent emotional feedback) (cf. Sha & Jiang, 2016).

Of note, many studies of value-driven attentional capture, whether employing monetary reward (Anderson & Halpern, 2017; Anderson et al., 2011; Cho & Cho, 2021; Qi et al., 2013; Sha & Jiang, 2016) or social reward such as emotional faces (Anderson, 2016, 2017; Anderson & Kim, 2018; Kim & Anderson, 2020), have exclusively used red or green as target colors during the training (association) phase and as distractor colors during the test phase. However, these studies have made no attempt to differentiate between the effects of the color itself and the association of the color with reward or emotion induced in the laboratory. Instead, the reported effects of the experimental manipulation are usually averaged across the two colors, assuming their equivalence. However, recent investigations of color–emotion preferences (Jonauskaite et al., ) have found that certain colors, such as red, are often linked with specific emotions like anger or love, while others, such as green or purple, lack such associations. Additionally, red is typically preferred over green in spontaneous selection paradigms, although this preference can be changed by the emotional context (Maier et al., 2009). Thus, it remains unclear whether the color-reward/emotion manipulations employed in previous studies actually elicit the same effect with both colors. While this may be of lesser importance in studies examining monetary reward associations, random color–emotion associations and averaging results across participants may obscure underlying color–emotion preferences and their interaction with the color–emotion associations. Accordingly, it is crucial to examine the effects of the emotion association separately for the critical colors—an approach we adopted in the present study.

Moreover, it is unclear to what extent interference caused by emotion-associated distractors depends on the strength of the individual’s association between a specific color and emotional feedback during the association phase. Previous research has primarily focused on average differences between the reward/emotion-related distractor conditions rather than the learning vector underlying reward/emotion-driven attentional capture. However, it is likely that the attentional interference displayed by an observer in the test phase is positively correlated with the strength of the reward/emotion association during the association phase. Participants who establish a stronger association between a target color and a non-neutral emotion stimulus (e.g., a happy face) during the training phase are expected to exhibit a greater difference in interference between the two types of emotion-associated color distractor in the test phase (non-neutral vs. neutral). To address this, in the present study, we analyzed the mean differences in interference between the two types of emotion-associated distractors in the test phase. Additionally, we conducted a correlation analysis using the individual learned emotion association during the training phase as a predictor of the differential valence-dependent interference effect observed in the test phase.

To this end, we conducted three experiments using a modified version of the value-driven attentional-capture paradigm inspired by Kim and Anderson (2020). In the association phase, participants searched for a red or green target circle among non-target circles with heterogeneous colors. Upon providing a correct response, they received feedback in the form of facial–emotional expressions (Experiments 1 and 3) or emotional scenes (Experiment 2). In the test phase, participants had to detect a shape pop-out target, specifically, a diamond among non-target circles. The display items were heterogeneous in color, with one of the items potentially having an emotion-associated color (red or green). In the test phases of Experiments 1 and 2, the two emotion-associated colors were entirely irrelevant to the search task; they were only implemented in a non-target item (i.e., the ‘distractor’) to examine differential attentional-interference effects, following Kim and Anderson’s (2020) approach. Conversely, in the test phase of Experiment 3, the emotion-associated colors were implemented in the search ‘target’ to investigate differential attentional facilitation effects. For the correlation analysis, we defined the valence preference as the difference in RT between the neutral- and pleasant-associated targets during the training session and the valence-dependent effect as the RT difference between the neutral- and pleasant-associated distractors (Experiments 1 and 2) or targets (Experiment 3) during the test session.

## Experiment 1

Experiment 1 aimed to re-examine whether a task-irrelevant color—red or green, previously linked to either happy or neutral emotional feedback—would impede, and do so variably, visual search for a singleton-shape target (a diamond among circles). In Kim and Anderson’s (2020) study, the emotional valence (high or low) carried by facial feedback did not influence search accuracy or response speed during the association phase. However, during the subsequent test phase, Kim and Anderson observed distractor interference: slowed RTs when the distractor was present relative to absent. Importantly, they did not observe any differences between the two distractor conditions linked to pleasant and neutral emotional feedback. Of note, however, the search displays in the test phase were presented for a rather long duration (1800 ms). Here, we tested both short- (300 ms) and long- (1500 ms) duration search displays in separate sessions. We included the short-duration displays on the premise that, if task-control mechanisms are stressed by the need for rapid processing, brief exposures might more effectively force variations in attentional capture owing to emotional valence. When perceptual and response decisions need to be made quickly, distractors might command attention relatively automatically, because any reactive control processes (Geng, 2014) aimed at reducing distractor interference might activate too late to impact stimulus-driven attention allocations (see also Sauter et al., 2021).

Another point of potential significance, as previously noted, is the tendency to overlook specific colors associated with different rewards in previous studies employing the value-driven attentional-capture paradigm (Anderson & Halpern, 2017; Anderson & Kim, 2018; Anderson et al., 2011; Cho & Cho, 2021; Kim & Anderson, 2020; Qi et al., 2013; Sha & Jiang, 2016). Given the potential interaction between color and emotion (e.g., Jonauskaite et al., 2019a, 2019b, 2020), we expressly included ‘color’ as a factor in our statistical analyses (even though we counterbalanced the specific color-to-valence associations across participants). As argued in the Introduction, this approach may reveal color–valence preferences that might otherwise remain undetected in standard analyses. Accordingly, we included a correlation analysis between the color–valence preference in the association phase and the valence-dependent effect in the test phase.

### Method

#### Participants

A total of 36 (16 males) healthy university students (Mean age = 29.03, *SD* = 4.19 years) participated in Experiment 1. The number of participants was based on sample sizes employed in the previous studies (Anderson, 2017; Kim & Anderson, 2020), ranging from 24 to 28. We increased the sample size to 36 for the online experiment. With the default *α* = 0.05 and the power $\beta$ = 0.8, the required effect size for the valence association to be detected is medium, ${d}_{z}$= 0.48. Similarly, the effect size required for the correlation analysis is also medium ${f}^{2}$ = 0.231.

Participants were recruited through a public announcement. Exclusion criteria for participation were (self-reported) diagnosis of a neurological or psychiatric disorder or, respectively, color blindness. Upon recruitment, detailed experimental instructions were sent to each participant via email, and then, participants accessed an online (experimental) platform, “Pavlovia”, to perform the experiment. Participants signed informed consent prior to the experiment and were compensated for their service at a rate of 9 Euro per hour. The study was approved by the Ethics Board of the LMU Faculty of Psychology and Educational Sciences.

#### Apparatus and stimuli

We used the free open-source cross-platform “PsychoPy3” (Peirce et al., 2019) for stimulus presentation and data collection, while the experiment was run via the online platform “Pavlovia” (https://pavlovia.org). Participants were instructed to be alone in a quiet room, adopting a viewing distance of 60 cm from the monitor. The screen size was 13.3 inches, as confirmed by all participants.

During the *association phase*, the search display consisted of a ring (radius of 5.4° at the viewing distance) of eight circles spaced equidistantly from the central fixation cross (circle: 2° in diameter), each with an oriented white bar (subtending 1.6° × 0.3°) inside. The response-relevant ‘target’ item was either a single green or red circle, whereas the remaining seven non-target circles were of different non-repeating (i.e., heterogeneous) colors, randomly selected from blue, yellow, cyan, magenta, silver, gray, olive, purple, teal, and navy.^1^ None of the non-target colors appeared twice within a given trial display. The target was equally likely to appear at any of the eight ring positions (i.e., target location was randomized across trials). The bar inside the (red or green) target circle was either vertical or horizontal, whereas the bars in the non-target circles were randomly tilted 45° to the left or the right. The feedback pictures were 58 photographs of happy faces and 58 of neutral faces (50% female and 50% male faces), selected from the Karolinska Directed Emotional Faces picture set (Lundqvist et al., 1998). Following Kim and Anderson (2020), happy (pleasant-valence) and neutral (neutral-valence) faces were used as social-reward feedback for correct responses in the association phase. Happy faces are considered to be of high social-reward magnitude, as compared to (low-magnitude) neutral faces.

During the *test phase*, the search display consisted of a ring composed of seven non-target circles plus a singleton-shape diamond, the response-relevant target, in heterogeneous colors; the circle and diamond stimuli subtended 2° of visual angle, and the set of possible colors was the same as in the association phase. The bars in the non-targets were again either left- or right-tilted, while the bar in the target was either vertical or horizontal. Importantly, while the non-target circles were of different colors, one circle could be red or green (i.e., possess the color of one of the targets in the association phase). The target was never red or green.

#### Procedure

During the initial association phase (Fig. 1a), each trial started with a central fixation cross (subtending 1° × 1° of visual angle) for 1000 ms. This was succeeded by a search display of 300 ms, which was immediately masked by a dot mask (subtending 6.4° × 6.4°) for another 300 ms. Participants were instructed to fixate on the central cross and, once the search display appeared, identify the orientation of the bar within the target circle by pressing “J” with their right-hand index finger for vertical orientation or “F” with their left-hand index finger for horizontal one, as quickly and accurately as possible. The display appeared for the full exposure duration unless the participant responded before 300 ms had passed, causing the display to be immediately terminated by the mask. A correct response prompted a 3 s display of a photograph inducing either a pleasant or a neutral emotion, as depicted in Fig. 1d and e. An incorrect response was followed by a 3 s “Incorrect” notification. Participants were instructed to view the content of the feedback photographs attentively. The target color (either red or green) was consistently associated with the valence of the feedback photograph (either pleasant or neutral) within each participant, but counterbalanced across participants. Trials were separated by an inter-trial interval (ITI) of 500 ms. This phase featured 348 trials, divided into four blocks of 87 trials each, with every feedback face appearing thrice. Participants decided the length of the short breaks between these blocks.

After the association phase (Fig. 1b), participants took a short break before proceeding to the test phase. Each trial began with a central fixation cross for 1000 ms, followed by a search display. The search display had a duration of either 300 ms (short-exposure block) or 1500 ms (long-exposure block) in different trial blocks, and was then superseded by a 300 ms dot-mask. The display remained for the full duration or until a response was made. Participants were told to maintain fixation on the central cross during the search-display presentation and, regardless of the display items’ color,^2^ find the target diamond and report the orientation of the bar inside it by pressing “J” for the vertical and “F” for the horizontal, as fast and accurately as possible. An “Incorrect” warning displayed centrally for 500 ms followed inaccurate responses, with correct responses yielding no feedback. The next trial began after an ITI of 500 ms.

Crucially, in half of the test trials, one non-target circle, referred to as the ‘distractor’, was colored either red or green, which was previously linked to either happy or neutral faces during the association phase (distractor-present trials); the displays in the remaining trials contained no distractors, that is, there were no non-target circles colored red or green. On distractor-present trials, the emotion-associated distractor circle could equally be red or green. The test phase was divided into two subsections, each featuring a different display exposure, one shorter and one longer, counterbalanced across participants. Each subsection consisted of four trial blocks, each comprising 70 trials, yielding a total of 560 trials in the test phase. Both the association and test phases were preceded by some practice trials (10 trials for the association phase and 15 trials for each subsection of the test phase) to familiarize participants with the task, which were excluded from further statistical analysis. The whole experiment lasted about one and a half hours.

#### Data processing and statistical analysis

Practice trials, trials with response errors, and trials with RTs shorter than 150 ms or longer than 3000 ms (on average, 2% and, respectively, 2% of the total trials) were excluded from further analysis. We used linear mixed models to analyze the data, considering Valence Association, Exposure Duration, and Distractor Color as fixed factors, and carried out ANOVA tests for those fixed effects and their interactions. In the test session, distractor-absent trials contained no valence-associated distractors. Therefore, the full-factorial model with Valence Association, Exposure Duration, and Distractor Color would yield multiple rank-deficient coefficients (i.e., unbalanced data). To avoid this, we excluded any interactions involving Distractor Color. For effect-size calculations, we employed Cohen’s *d* for contrast comparisons, and Cohen’s ${f}^{2}$. All statistical analyses were conducted using R (R Core Team, 2022). We used the “lme4” package (Bates et al., 2014), while the “emmeans” and “effect-size” packages (Ben-Shachar et al., 2020) were employed for comparisons and effect sizes, respectively. Bayes factors were computed from marginal likelihoods with the “brms” package (Bürkner, 2017), and reliability measures were estimated by the “split-half” package (Parsons, 2021).

### Results

#### Association phase

**Fig. 2:**
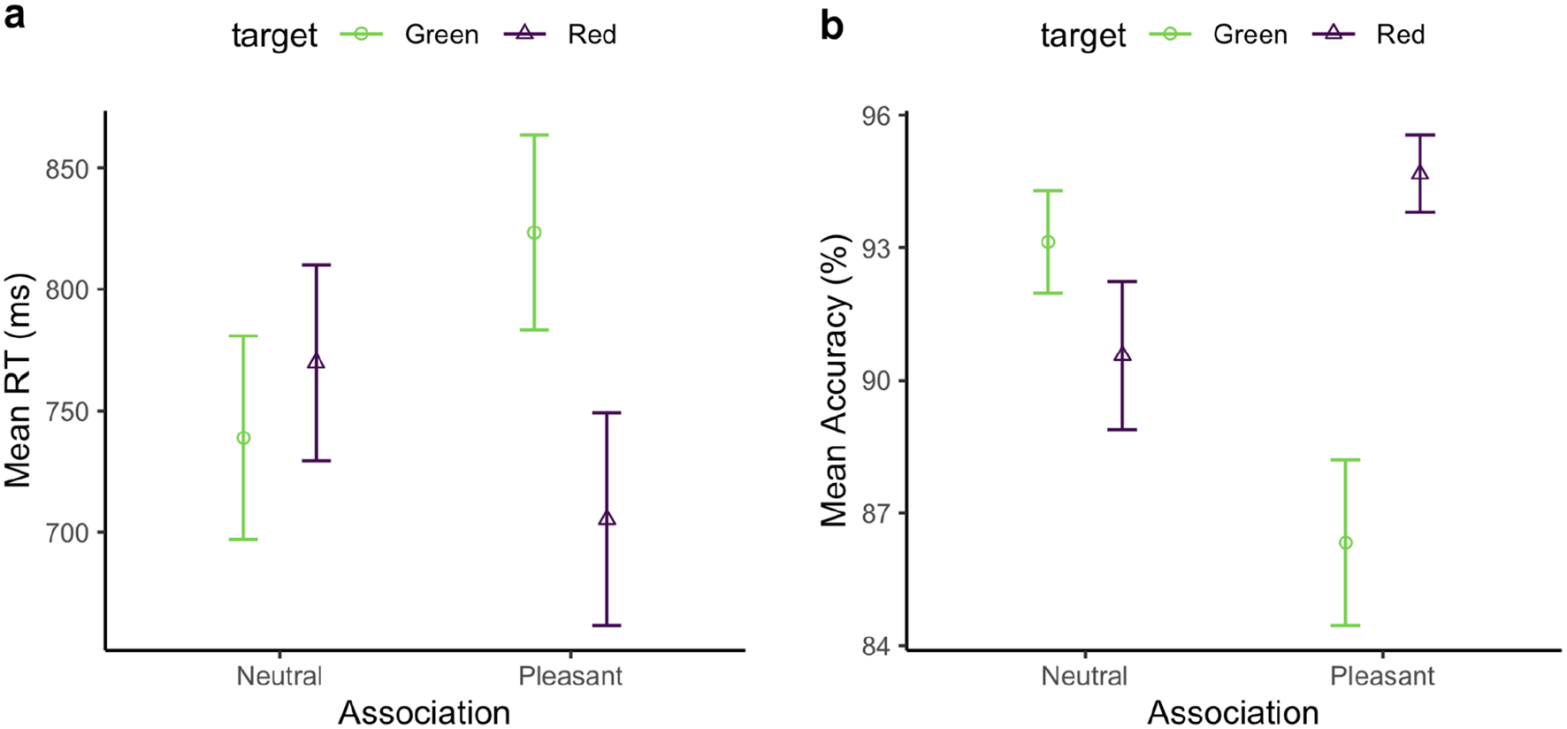
Mean RTs (**a**) and accuracies (**b**), and associated standard errors (SEs), for the facial-emotion association, separately for the green (circle) and red (triangle) targets in the association phase of Experiment 1

Performance accuracy was quite high overall (91.2%). An ANOVA with the factors Association and Target Color revealed the Target-Color main effect to be significant, *F*(1, 34) = 10.28, *p* < 0.01, ${\eta }_{p}^{2}=0.23$, but not the Association effect, *F*(1, 34) = 2.23, *p* = 0.145, ${\eta }_{p}^{2}= 0.06$. Accuracy was significantly higher for the red target vs. the green target. The interaction was also significant, *F*(1, 34) = 9.31, *p* < 0.01, ${\eta }_{p}^{2}= 0.22$, due to a marked discrepancy between two target colors in the pleasant association vs. the neutral association: accuracy was the highest when the red target was paired with a happy face (94.6%), and the lowest when the green target was associated with a happy face (86.0%; see Fig. 2).

The overall correct RT was 757 ms. Figure 2a shows the mean RTs for targets defined by colors associated with the neutral- and, respectively, pleasant-face feedback. An ANOVA examining the mean RTs with respect to the Target Color (red vs. green) and Valence association (neutral vs. pleasant) yielded a significant main effect of Target Color, *F*(1, 34) = 31.47, *p* < 0.001, ${\eta }_{p}^{2}= 0.48$. However, neither the main effect of Valence, *F*(1, 34) = 1.67, *p* = 0.205, ${\eta }_{p}^{2}=0.05$, nor the Valence × Target–Color interaction were significant, *F*(1, 34) = 1.61, *p* = 0.213, ${\eta }_{p}^{2}=0.05$.

Thus, in the association session, participants responded to red targets faster and more accurately than to green targets. However, the response feedback, whether pleasant or neutral in valence, had no significant influence on search performance.

#### Test phase

The response accuracy was 90.5% overall, exhibiting mild fluctuations across all conditions (with rates ranging from 87.3 to 94.3%). An ANOVA of response accuracy with the factors Distractor Valence (neutral, pleasant, absent), Exposure Duration (short vs. long), and Distractor Color revealed only a significant main effect of Exposure Duration, *F*(1, 174) = 105.3, *p* < 0.001, ${\eta }_{p}^{2}= 0.29$. No other (main or interaction) effects were significant, with *F*s < 0.59, *p*s > 0.55. Response accuracy was significantly lower for the short- (87.9%) vs. the long-display presentations (93.6%).

Similarly, an ANOVA of the correct RTs also revealed only a significant main effect of Exposure Duration, *F*(1, 174) = 7.87, *p* = 0.006, ${\eta }_{p}^{2}= 0.04$. All other (main and interaction) effects were non-significant, with *F*s < 1.88, *p*s > 0.17. On average, the mean RT was faster with the short- (638 ms) vs. the long-exposure durations (654 ms). However, there was no evidence of a (significant) valence-associated distractor-capture effect.

#### Association/test-phase correlation

To delve deeper into the potential correlation between color–emotion association learning in the initial phase and distractor interference in the subsequent test phase on a group level, we first calculated individual observers’ *color–valence preference*, defined as the difference in RTs between target colors associated with pleasant vs. neutral-face feedback during the association phase. Positive preference scores indicate that responses were slower for the color associated with the pleasant emotion vs. the color associated with the neutral emotion, while negative scores signify faster responses to the pleasant- (vs. neutral-) associated colors. Using 5000 random splits, the Spearman–Brown corrected reliability estimate for the color–valence preference was quite high, *r* = 0.86 with 95% CI [0.76, 0.93]. Similarly, we calculated the corresponding individual distractor-effect scores, defined as the RT difference between pleasant and neutral distractor conditions, during the test phase. A 5000 random-sample split-half reliability analysis indicated that the Spearman–Brown coefficients were only of moderate-to-low strength (0.34 with 95% CI [− 0.08, 0.64]) for the long exposure, and of low strength (0.17 with 95% CI [− 0.35, 0.59]) for the short exposure.

**Fig. 3:**
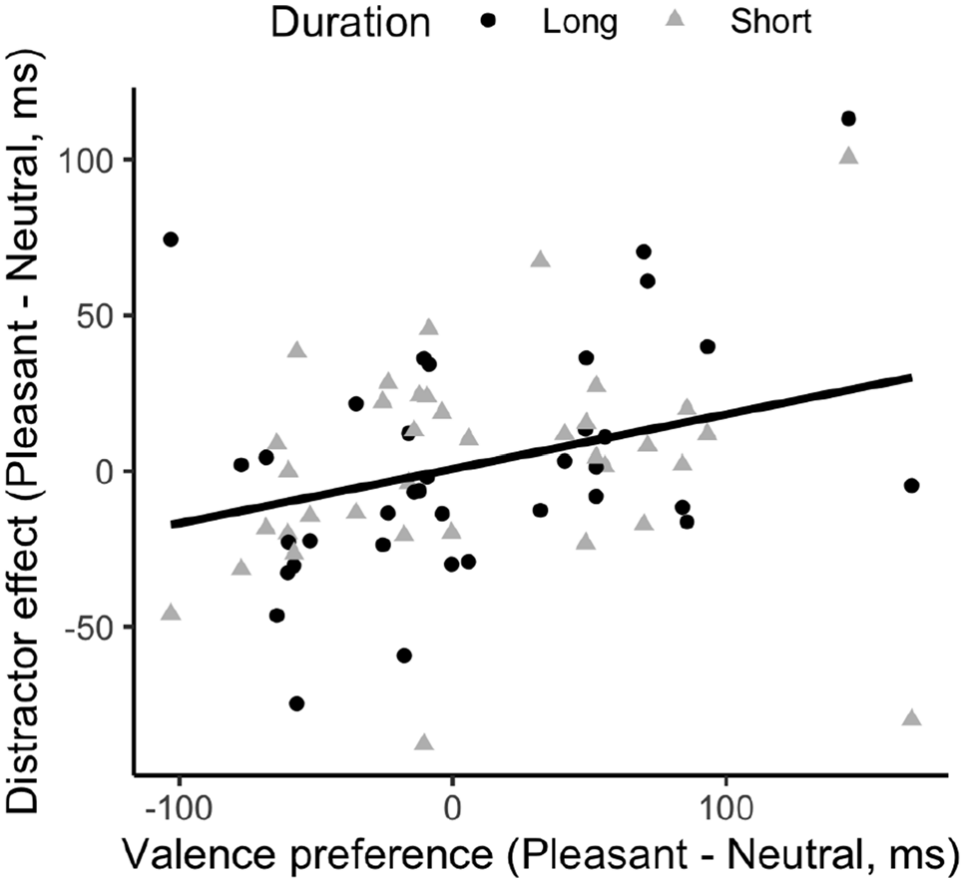
Correlation between the color–valence preference in the association phase and the distractor (interference) effect in the test phase, across the long- (circle) and short- (triangle) duration sessions

Subsequently, we assessed the correlation between the individual valence-preference scores and the corresponding distractor-effect scores. Given that Exposure Duration was a significant factor in the test phase, we calculated the distractor effect separately for the short- and long-exposure durations. Figure 3 depicts the correlation between the color–valence preference and the distractor effect. The correlation was significant, with a coefficient of *r* = 0.3, *p* = 0.01, and a significant positive slope (0.174, *p* = 0.01). In essence, participants’ color–valence preference remained consistent across the association and test phases.

### Discussion

The results of Experiment 1 indicated that, in the initial association phase, the response speed was primarily influenced by the target color itself, with little impact of the emotional-face feedback. This absence of a substantial effect of the emotional feedback on RTs aligns with Kim and Anderson (2020), who also found no evidence of a color/social-reward association. Of note, the lack of a reliable effect is not uncommon in studies using social rather than monetary-reward feedback (Anderson, 2016; Anderson & Kim, 2018; Kim & Anderson, 2020).

Moreover, Experiment 1 failed to establish a valence-dependent modulation of attentional capture/interference in the average RTs during the test phase (as illustrated in Fig. 3). This lack of reliable differential capture/interference aligns with the results of Kim and Anderson (2020). However, contrary to our findings, Kim and Anderson (2020) did report a general emotion- or search-history-related attentional-capture/interference effect: significantly prolonged RTs in the presence, vs. the absence, of any (previously) reward-associated distractor. This is not reflected in our results, where only the presentation duration mattered.

Intriguingly, we found a positive correlation between the initial valence preference and the distractor-interference effect. Unlike the typical reward-based attentional capture/interference, where a high-reward-associated target color is detected faster in the association phase and then interferes more when introduced as a distractor color in the test phase, we found participants who took longer to respond to the ‘target’ color associated with pleasant emotions during the association phase to experience greater interference from this color ‘distractor’ in the test phase. This suggests a general underlying effect expressed as either facilitation or interference, likely reflecting the congruency between the color–valence pairings assigned during the association phase and participants’ individual color–valence preferences. Cultural factors, such as the association of red with pleasant emotions in Asian cultures, can influence these preferences. As color evaluations rely on established and stable mental color–emotion representations (e.g., Jonauskaite et al., 2019a, 2019b), our correlation findings suggest that congruence between color–valence pairings in the association phase and participants’ preferences facilitates performance. Incongruent pairings do not alter the individual preferences, which then influence performance in the test phase, contributing to the positive correlation that we found (Fig. 3c). It should be noted that the positive correlation was not inflated by the color preference per se: both congruent- and incongruent-pairing groups exhibited the same positive trend. Thus, the fact that the congruence of the color–valence pairing and participants’ own preference influenced RTs in the same direction, regardless of whether the critical color item was a target or a distractor, suggests that the presence of color that fits a participant’s color–valence preference may generally *facilitate* task performance. Nevertheless, the interpretation should be treated cautiously, given that the reliability measure obtained during the test session was only of moderate-to-low strength.

In short, Experiment 1 failed to find evidence of an emotional-reward feedback modulation of attentional capture/interference by task-irrelevant distractor colors. This might be due to the use of facial–emotional feedback stimuli in Experiment 1. As emotional scenes and facial expressions are neuro-cognitively processed differently (Britton et al., 2006) and emotional scenes offer better manipulation of valence and arousal dimensions, we introduced emotional scenes as reward feedback in Experiment 2 to re-examine the potential impact of emotional associations on attentional interference/capture.

## Experiment 2

Experiment 2 was designed to examine whether social-emotionally charged images would influence attentional interference/capture through valence-associative learning. Considering arousal as a critical dimension of emotional stimuli, we examined the impact of valence-dependent attentional-capture/interference following an association phase in which the alternative colors (red and green) were matched with either high- or low-arousal scenes in separate experimental sessions. Given that arousal can enhance memory consolidation of perceptually encoded, task-relevant visual items, we considered it potentially important to systematically vary the arousal (high vs. low) and valence (pleasant vs. neutral) values of our emotional-reward–feedback stimuli. And as evoked arousal may last beyond a single trial’s duration, we manipulated the arousal level in separate sessions to prevent possible contamination from one trial to the next. We hypothesized that if associating a particular target color with an emotional feedback scene would enhance search performance in the association phase, the corresponding color would then impede search performance when introduced as a distractor in the test phase. The impact might be enhanced by the arousal. Apart from the introduction of the arousal manipulation during the association phase (a within-subject manipulation) and social–emotional pictures, the design of Experiment 2 was the same as that of Experiment 1 in all other respects, allowing for a direct comparison.

Given the inherent limitations of the online format of Experiment 1, including the inability to ensure consistent display settings across participants, especially concerning color luminance, we conducted Experiment 2 in a laboratory setting to ensure more controlled experimental settings.

### Method

#### Participants

A new group of 38 (15 males) healthy university students was recruited (*Mean* age = 24.71, *SD* = 2.49). The sample size and the required effect size was the same as in Experiment 1. Participants provided informed written consent prior to starting the experiment and were compensated for their participation at a rate of 9 Euro per hour. The experiment was approved by the Ethics Board of the LMU Faculty of Psychology and Educational Sciences.

#### Apparatus and stimuli

Experiment 2 closely followed the design of Experiment 1, except that it was conducted in a sound-attenuated and dimly lit laboratory cabin. The monitor, calibrated for color (120 cd/m^2^ D65 white point), was a 24” TFT-LCD monitor (ASUS VG248QE, screen resolution 1920 × 1080 pixels, frame rate 120 Hz), positioned at eye level and a viewing distance of 60 cm from the participants. We used “PsychoPy3” (Peirce, 2008; Peirce & MacAskill, 2018; Peirce et al., 2019) for stimulus presentation and response recording. While the search displays remained the same as in the online Experiment 1, we had more control over the monitor settings in this on-site Experiment 2. All differently colored items in Experiment 2 were of a subjectively equal brightness level (as judged by the experimenters). Luminance measurements taken by a Chroma Meter (Konica Minolta CS-100A) revealed the items’ luminance to be closely matching: the luminance values of the red and green target/distractor items were 15.9 cd/m^2^ and 16.7 cd/m^2^, respectively, and while the other, differently colored non-target/non-distractor items averaged 18.7 (range 15.2–22.4) cd/m^2^; the black screen-background luminance was 0.14 cd/m^2^. Accordingly, the critical colors (red and green) did not stand out from the stimulus array by virtue of their luminance.

A total of 344 photographs depicting a social context (with one or more people), selected from the “IAPS” (Lang et al., 1997) and “EmoPics” (Wessa et al., 2010), were presented as social-reward stimuli. Selection of the IAPS stimuli was based on the pictures’ valence (neutral, pleasant) and arousal (low, high) values, yielding four sets, each of 86 pictures: neutral/low-arousal (mean valence: 5.16, arousal: 2.87), neutral/high-arousal (mean valence: 5.62, arousal: 3.56), pleasant/low-arousal (mean valence: 6.84, arousal: 4.41), and pleasant/high-arousal (mean valence: 7.11, arousal: 5.57).

#### Procedure

The procedure was generally the same as in Experiment 1, with a few differences. Emotional scenes (photos depicting pleasant and neutral social contexts) replaced happy and neutral emotional faces as reward feedback during the association phase (see examples in Fig. 1f and g). Additionally, the experiment was divided into two separate sessions: one featured low-arousal scenes and the other high-arousal scenes as reward feedback during the association phase, with both sessions using an equal distribution of 50% pleasant and 50% neutral photos. To avoid any potential carry-over effects, the experiment was divided into two sessions (low vs. high arousal), with a gap of 7–10 days in-between. The order of the sessions was counterbalanced across participants.^3^ Thus, the association of a target color with neutral vs. pleasant feedback was swapped across the high- and low-arousal sessions. No pleasant and neutral photos were repeated. Exposure time was handled in the same manner as in Experiment 1, with the search displays in the test phase appearing in short-exposure (300 ms) or long-exposure blocks (1500 ms), both terminated by the dot masks (displayed for 300 ms).

Each association phase consisted of 174 trials per session, totaling 348 trials across both sessions, with additional ten practice trials beforehand. The test phases consisted of 280 short-exposure and 280 long-exposure trials, totaling 1120 trials across both sessions, and additional ten practice trials as well. Thus, Experiment 2 contained double the number of trials as Experiment 1.

Upon completion of the experiment, participants were asked to rate the valence and arousal of all previously displayed photographs. The photographs were presented in random order for an unlimited exposure duration, with participants rating each photograph first for pleasure and then for arousal on a Likert-type 9-point scale (1 representing “very unpleasant” or “not aroused”, and 9 representing “very pleasant” and “very aroused”). An ANOVA of the ratings confirmed that our picture selection elicited the desired perception among participants: rated valence was higher for pleasant vs. neutral pictures: 7.03 vs. 5.61, *F*(1, 36) = 53.65, *p* < 0.001, ${\eta }_{p}^{2}$ = 0.6; and rated arousal was higher for high- vs. low-arousal pictures 3.67 vs. 3.12, *F*(1, 36) = 5.45, *p* = 0.036, ${\eta }_{p}^{2}$ = 0.13. Their interaction was also significant, *F*(1, 36) = 21.03, *p* < 0.001, ${\eta }_{p}^{2}$ = 0.37, owing to a lack of an effect of low vs. high arousal for neutral pictures (1.94 vs. 2.49).

### Results

#### Association phase

**Fig. 4:**
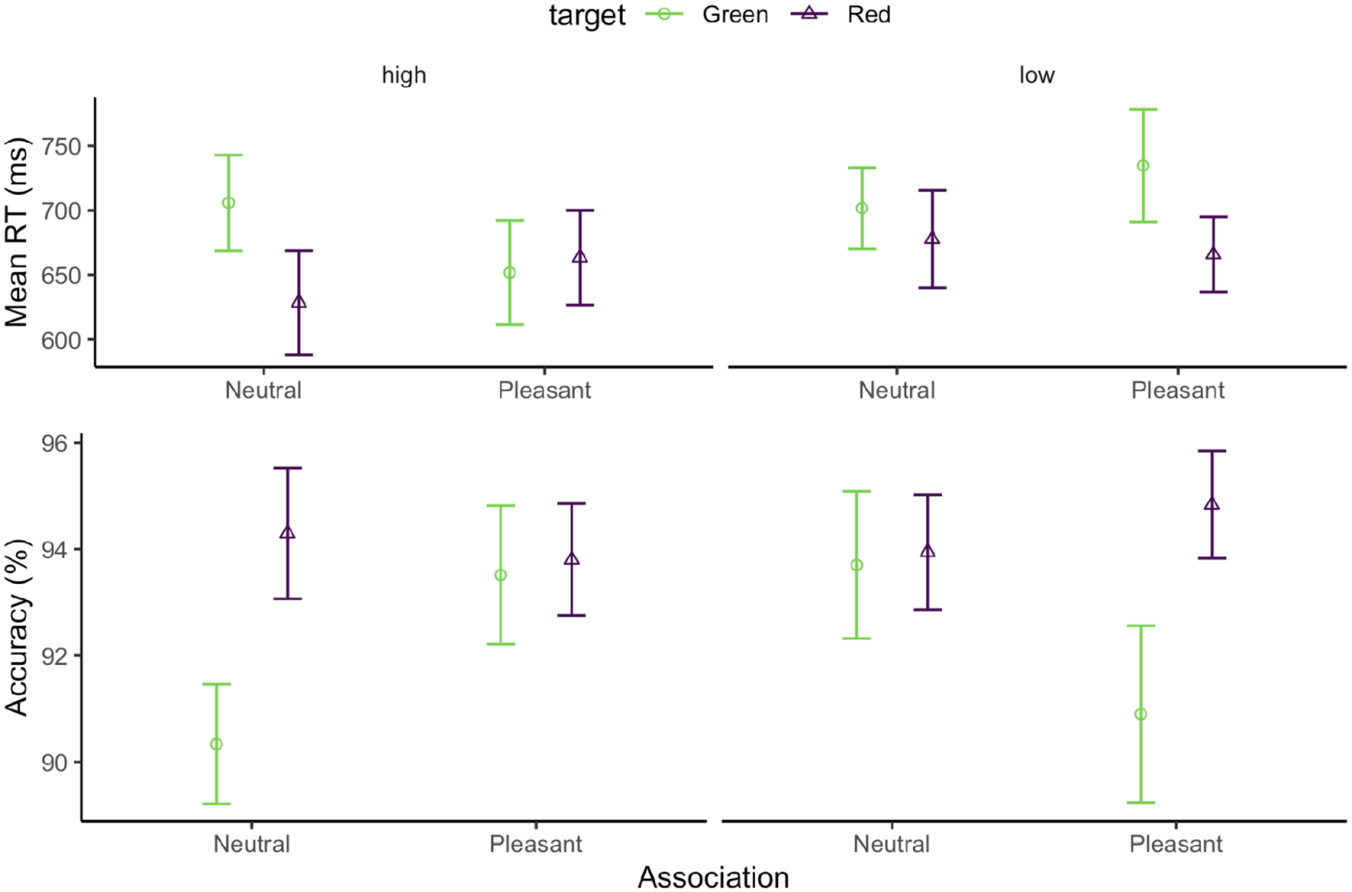
Mean RTs (the upper panel) and accuracies (the lower panel), and associated standard errors (SEs), for the emotional association, separately for the green (circle) and red (triangle) targets, and the high- (left panel) and low- (right panel) arousal sessions, in the association phase of Experiment 2

Response accuracy was 93.2% overall, appearing comparable across all conditions. The mean accuracies are shown in the lower panel of Fig. 4. An ANOVA of response accuracy with the factors Valence (neutral, pleasant), Arousal (high, low), and Target Color (red, green) revealed only a significant main effect of Target Color, *F*(1, 108) = 11.43, *p* = 0.001, ${\eta }_{p}^{2}$ = 0.1. The interaction between Valence and Arousal was marginally significant, *F*(1, 108) = 3.40, *p* = 0.067, ${\eta }_{p}^{2}$ = 0.03. All other (main and interaction) effects were non-significant, with *F*s < 1.73, *p*s > 0.19. On average, the red target yielded higher accuracy.

The mean correct RTs are shown in the upper panel of Fig. 4. A separate three-way ANOVA of the RTs revealed significant main effects of Target Color, *F*(1, 108) = 7.78, *p* = 0.006, ${\eta }_{p}^{2}$ = 0.07, and of Arousal, *F*(1, 108) = 5.27, *p* = 0.023, ${\eta }_{p}^{2}$ = 0.05. RTs were overall faster in the high-arousal (662 ms) vs. the low-arousal session (695), and faster when the target was red (659 ms) vs. green (699 ms). There were no (main or interaction) effects involving Valence, *F*s < 0.6, *p*s > 0.43. Thus, pleasant vs. neutral emotional-scene feedback did not significantly modulate the RTs to the associated color-defined targets in Experiment 2.

#### Test phase

We applied similar linear-mixed-model analyses as those used in Experiment 1 here, with the fixed factors Arousal (high, low), Exposure Duration (short, long), Distractor Valence (absent, neutral, pleasant), as well as Distractor Color (red, green). Any interactions with Distractor Colors were excluded due to unbalanced data (given distractor-absent displays did not contain any red or green colors). With 93.5%, the overall response accuracy was quite high in the test phase. The analysis of response accuracy revealed a significant main effect of Exposure Duration, *F*(1, 406) = 149.6, *p* < 0.001, ${\eta }_{p}^{2}$ = 0.27. Accuracy was significantly higher with long- (95.7%) vs. short-exposure durations (91.2%). The main effect of Arousal was marginally significant, *F*(1, 406) = 3.6, *p* = 0.058, ${\eta }_{p}^{2}$ = 0.01, with slightly higher accuracy in the high- (93.8%) vs. the low-arousal session (93.1%). No other (main or interaction) effects were significant, *F*s < 1.87, *p*s > 0.154.

A similar analysis of the correct RTs revealed significant main effects of Arousal, *F*(1, 406) = 23.6, *p* < 0.001, ${\eta }_{p}^{2}$ = 0.05, and Duration, *F*(1, 406) = 12.97, *p* < 0.001, ${\eta }_{p}^{2}$ = 0.03. RTs were, on average, faster in the high- (556 ms) vs. the low-arousal session (586 ms), and faster in the short (560 ms) vs. the long-exposure-duration condition (582 ms). No other effects were significant, *F*s < 0.898, *p*s > 0.343. Similar to the findings in Experiment 1, we failed to find any modulation of response speed by the valence-associated distractor.

#### Association/test-phase correlation

Similar to Experiment 1, we conducted a correlation analysis between the color–valence preference in the association phase and distractor interference in the test phase. As the main factors of Arousal and Exposure Duration significantly influenced RTs, we evaluated the distractor effect separately for their combinations. Using 5000 splits, the Spearman–Brown corrected reliability estimates for the color–valence preference score during the training session were of medium strength, *r* = 0.41 with 95% CI [0.2, 0.58] for the high-arousal session, and *r* = 0.34 with 95% CI [− 0.07, 0.62] for the low-arousal session. An analogous analysis for the test session revealed the reliability measures to be relatively low: 0.22 [− 0.06, 0.48], 0.07 [− 0.34, 0.44], − 0.13 [− 0.48, 0.29], and 0.31 [− 0.15, 0.63] for the high-arousal/long exposure, high/short, low/long, and low/short conditions, respectively.

**Fig. 5:**
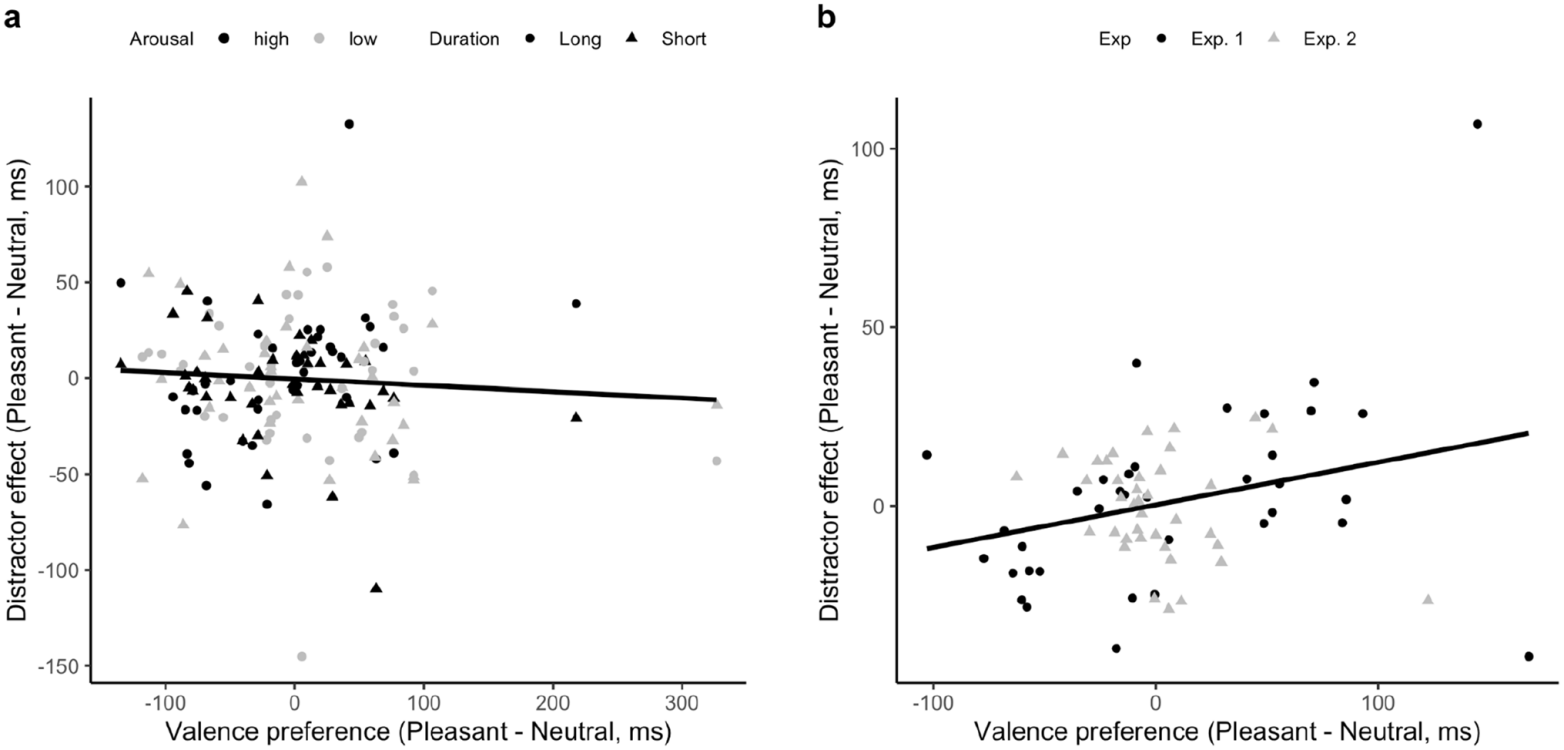
**a** Correlation between the color–valence preference in the association phase and the distractor (interference) effect in the test phase, across the high- (black) and low- (gray) arousal sessions and the long- (circle) and short- (triangle) exposure conditions. **b** Correlation between the color–valence preference in the association phase and the distractor (interference) effect in the test phase, across Exp. 1 (circle) and Exp. 2 (triangle)

Figure 5 depicts the relation between the valence preference and the distractor-interference effect. In contrast to Experiment 1, Experiment 2 failed to show any significant correlation: *r* = − 0.069, *p* = 0.397. Given that Experiments 1 and 2 shared essentially the same experimental paradigm, except for the type of emotional picture presented as reward feedback in the association phase, we also conducted the correlation analysis for both experiments combined. For this, we collapsed the factor Duration in Experiment 1 and both the factors Arousal and Duration in Experiment 2, so that each participant represents a single point. This combined analysis yielded a statistically robust positive correlation: *r* = 0.28, *p* = 0.018, with a linear slope of 0.119.

### Discussion

Similar to Experiment 1 (which used emotional-face feedback), Experiment 2 (which used emotional-scene feedback) failed to establish reliable effects of the emotional-reward valence associated with a particular target color in the association phase and the respective distractor color in the test phase. Although the correlation analysis failed to show significance, pooling the two experiments together still revealed a positive correlation between participants’ color–valence preference in the association phase and distractor interference in the test phase.

## Experiment 3

In a standard value-driven *attentional-capture* paradigm, the color stimuli associated with different value levels are introduced exclusively as distractors in the test phase. These colors are never the target color. As seen in our Experiments 1 and 2, differential effects between distractor colors associated with pleasant vs. neutral emotions have proven difficult to demonstrate. Conceptually, an enhanced ‘priority’ signal from a value- or emotion-associated color should also be observable when the color occurs at the target location. In this scenario, the enhanced color signal would merge with the signal highlighting the target in the shape dimension, that is: the value-/emotion-associated color signal would summate with the shape signal, amplifying the attentional priority of the target by way of a redundancy gain (e.g., Krummenacher & Müller, 2014; Krummenacher et al., 2001, 2002; Nasemann et al., 2023) and so expediting the allocation of attention to the target location. This may be so especially when ‘color’ is potentially helpful in detecting and localizing the target—as opposed to when it is only distractive, as in the standard *involuntary*-attentional-capture paradigm. In such cases, ‘color’ might be effectively ignored as irrelevant through top–down dimensional set (e.g., Müller et al., 2009)—also referred to as ‘second-order feature suppression’ by Gaspelin and Luck (2018) and Won et al. (2019). In contrast, ‘color’ might be construed as helpful, or relevant, if the value-/emotion-associated color is more likely to occur at the location of the (shape-defined) target, rather than that of a non-target location.

On this background, we hypothesized that a valence-associated color effect might be more evident under test-phase conditions in which the colors associated with pleasant vs. neutral emotions are statistically biased to appear at the target location (as compared to when they never occur at the target location, as in Experiments 1 and 2). To test this prediction in Experiment 3, we employed the same neutral- and pleasant-face photographs as social-reward stimuli in the association phase as in Experiment 1. For the aforementioned reasons, we expected valence-dependent facilitation of the RTs to the target—in particular, because participants have less incentive to down-modulate color processing when an emotion-associated color appears in the target, as opposed to when it appears in a distractor. In the latter scenario, even singleton signals from the (irrelevant and potentially interfering) color dimension can be relatively efficiently excluded from priority computations, especially when they occur frequently (e.g., Geyer et al., 2008; Liesefeld et al., 2019; Müller et al., 2009; Won et al., 2020).

On the other hand, if our hypotheses from the discussion of Experiments 1 and 2 hold, namely, that observers’ pre-existing color–valence preferences impede the establishment of experimentally manipulated color–valence associations, we may not observe any valence-modulated effects of the critical colors, even if these occur in the target (as in Experiment 3) rather than a distractor (Experiments 1 and 2).

### Method

#### Participants

A new group of 25 university students participated in Experiment 3 for monetary compensation (12 males; *mean age* = 25.46, SD = 2.79, years). Given that the large sample size in Experiments 1 and 2 yielded similar effect sizes as previous studies (significant differences between the distractor-absent condition and distractor-present conditions, but not between high- and low-valence conditions), we used a similar sample size of 25 as employed in the previous study (Kim & Anderson, 2020). Protocols were the same as for Experiments 1 and 2.

#### Apparatus and stimuli

Experiment 3 was conducted in the same sound-attenuated and dimly lit laboratory cabin and used the same monitor as in Experiment 2. The specifications of the stimuli paralleled those in Experiment 1, with one key difference in the test phase: the color of the singleton diamond (i.e., the target) was either red (33% of the time), or green (33% of the time), or one of the other colors (i.e., blue, yellow, cyan, magenta, silver, gray, olive, purple, teal, and navy, collectively representing 33% of the total). None of the colors were replicated within the same trial display. Importantly, a non-target circle never appeared in the red or green color that was associated with the emotional-face feedback during the association phase. The luminance values of the search items were identical to those in Experiment 2.

#### Procedure

The procedure mirrored that of Experiment 1, with one key difference: in the test phase, the target colors from the association phase (red or green) were not assigned to the target item (diamond) in two-thirds of the trials. The target was consistently singled out by being the only diamond shape among homogenous circle shapes. In contrast, the color of the diamond item was essentially inconsequential to the task—in fact, it could not possibly aid performance in one third of the trials. Accordingly, participants were told to search for and respond to the diamond-shaped target, ignoring the colors.

### Results

#### Association phase

**Fig. 6:**
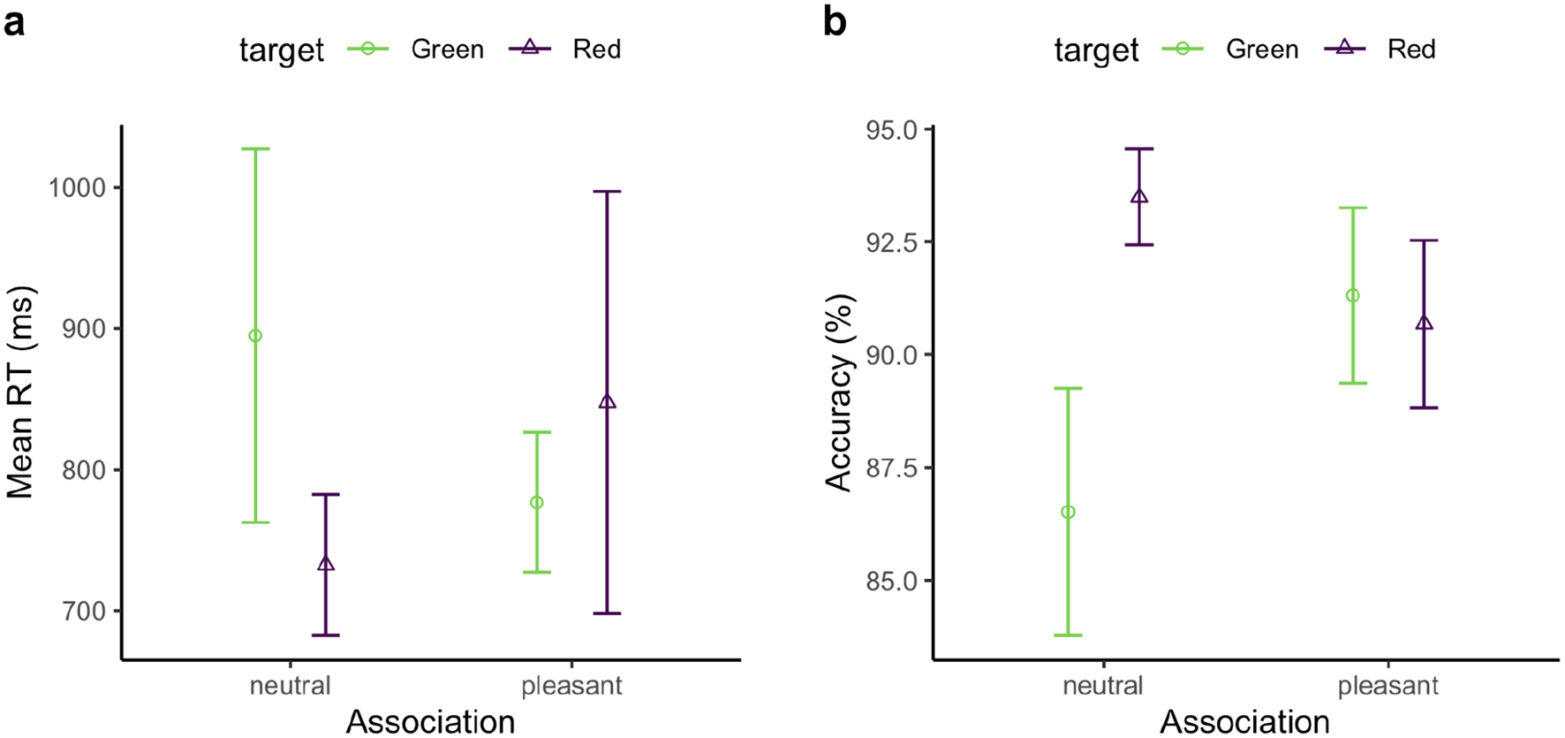
**a** Mean correct RTs (**a**) and accuracies (**b**), and associated standard errors (SEs), for the facial-emotion association, separately for the green (circle) and red (triangle) target in the association session of Experiment 3

Overall response accuracy was 90.4%, ranging from 86.2% for the green-neutral target to 93.4% for the red-neutral target. An ANOVA with the factors Target Color (red vs. green) and Valence Association (neutral vs. pleasant) revealed the Target-Color main effect to be significant, *F*(1, 23) = 6.35, *p* = 0.019, ${\eta }_{p}^{2}$ = 0.22; there were no (main or interaction) effects involving Valence Association, *F*s < 2.37, *p*s > 0.13 (Fig. 6). Again, accuracy was higher for the red (92.1%) vs. the green target (89%).

The mean correct RTs are shown in Fig. 6a. An analogous ANOVA of the mean RTs yielded a significant main effect of Target Color, *F*(1, 23) = 6.19, *p* = 0.02,${\eta }_{p}^{2}$ = 0.21. Neither the main effect of Valence nor the Valence × Target-Color interaction turned out significant, *F*s < 0.66, *p*s > 0.42.

Thus, in the association phase, participants responded to red targets faster and more accurately than to green targets. However, the feedback, whether pleasant or neutral in valence, did not significantly influence search performance. This pattern corroborates the findings of Experiments 1 and 2.

#### Test phase

**Fig. 7:**
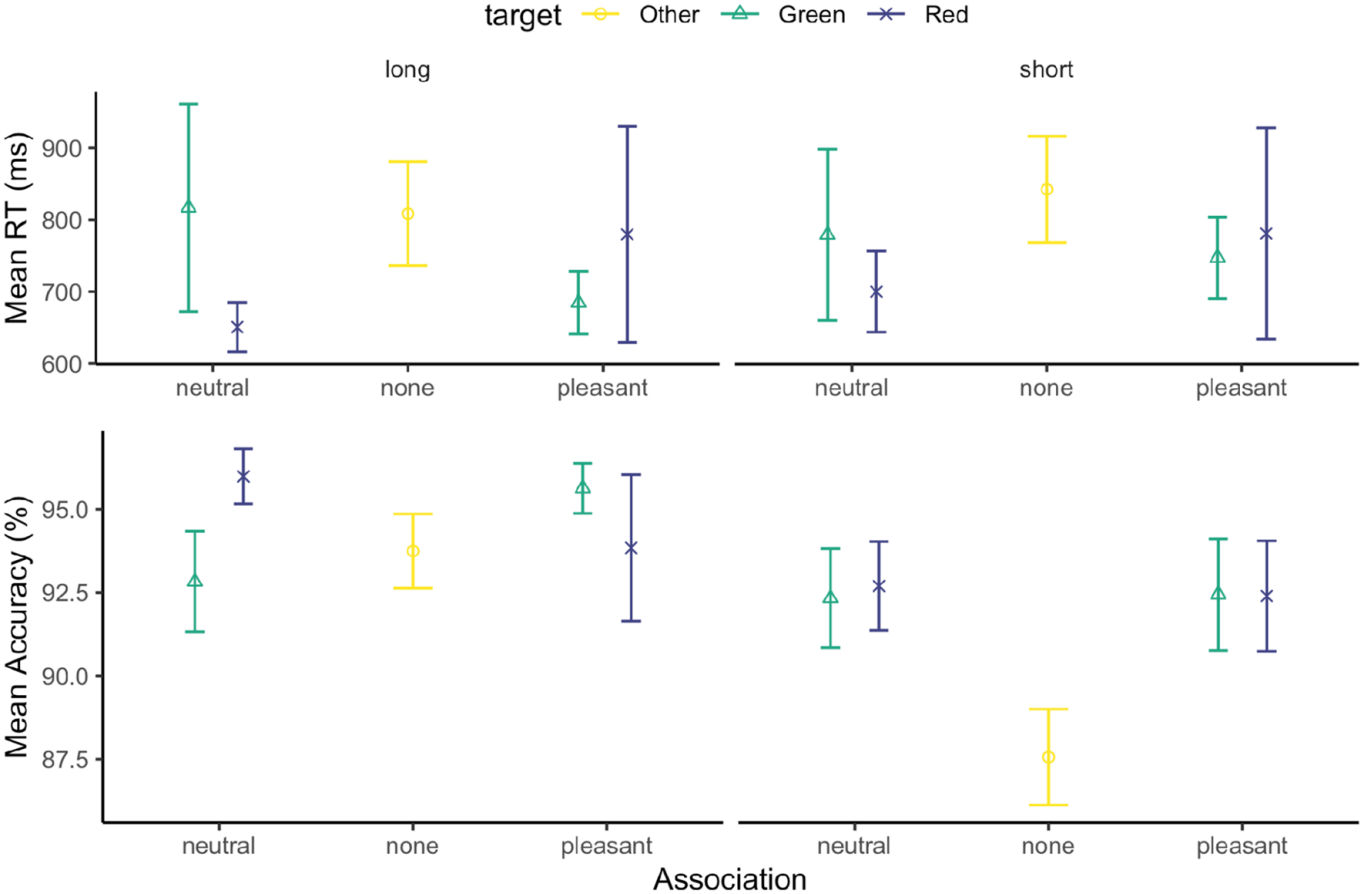
**a** Mean RTs and **b** accuracies, and their associated standard errors (SEs), separately for Valence Association (none, neutral, pleasant), Target Color (other, green, red), and Exposure Duration (long, short) conditions in the test session

Overall response accuracy was 92.5% in the test phase, ranging from 87.6% for the non-associated color target with short-display exposure to 95.9% for the neutral-red target with long exposure. We carried out a linear-mixed-model analysis of response accuracy, considering the fixed factors Exposure Duration (short, long), Valence Association (absent, neutral, pleasant), Target Color (red, green, other), as well as the interaction between Exposure Duration and Valence Association. The analysis revealed significant main effects of Exposure Duration, *F*(1, 119) = 24.09, *p* < 0.001, ${\eta }_{p}^{2}$ = 0.17, and Valence Association, *F*(2, 119) = 6.33, *p* = 0.002, ${\eta }_{p}^{2}$ = 0.10, but not of Target Color, *F*(1, 119) = 0.234, *p* = 0.63, ${\eta }_{p}^{2}$ < 0.01. Accuracy was reduced with short- (90.8%) vs. long-display exposures (94.3%). And post hoc comparisons revealed the main effect of Valence Association to be mainly due to accuracy being lowest for the non-associated target, compared to the neutral and pleasant-associated targets (*p*s < 0.011), while there was no difference between the latter (neutral vs. pleasant, *p* = 0.99). Accordingly, the Valence-Association effect is attributable to a general history effect, as the neutral- and pleasant-associated color targets had already been encountered as search targets during the association phase. The interaction between Valence Association and Exposure Duration was also significant, *F*(2, 119) = 3.597, *p* = 0.03, ${\eta }_{p}^{2}$ = 0.06, mainly owing to accuracy being lowest for the non-associated target in the short-exposure condition (Fig. 7b).

An analogous analysis of the mean RTs revealed the main effect of Valence Association to be significant, *F*(2, 119) = 18.07, *p* < 0.001, ${\eta }_{p}^{2}$ = 0.23. As disclosed by post hoc comparisons, this effect was mainly due to RTs being slowest for the non-associated target compared to the neutral- and pleasant-associated targets (825 ms vs. 734 and 747 ms, *p*s < 0.001), while the latter did not differ significantly (*p* = 0.84). Again, the substantial RT gains for the associated (red- and green-colored) targets (including the narrowing of the difference between the association- and test-phase RTs) are likely reflecting a target-history effect (Sha & Jiang, 2016): facilitated responding deriving from the valence-associated but task-irrelevant colors having already been encountered as target colors in the association phase. Of note, this history effect was itself not modulated by the color–valence association (if anything, RTs were slower for the pleasant- vs. the neutral-associated target: 747 ms vs 734 ms, n.s.). The main effects of Exposure Duration and Target Color, and the interaction between Valence Association and Exposure Duration were all non-significant, *F*s < 3.33, *p*s > 0.07.

#### Association/test-phase correlation

**Fig. 8:**
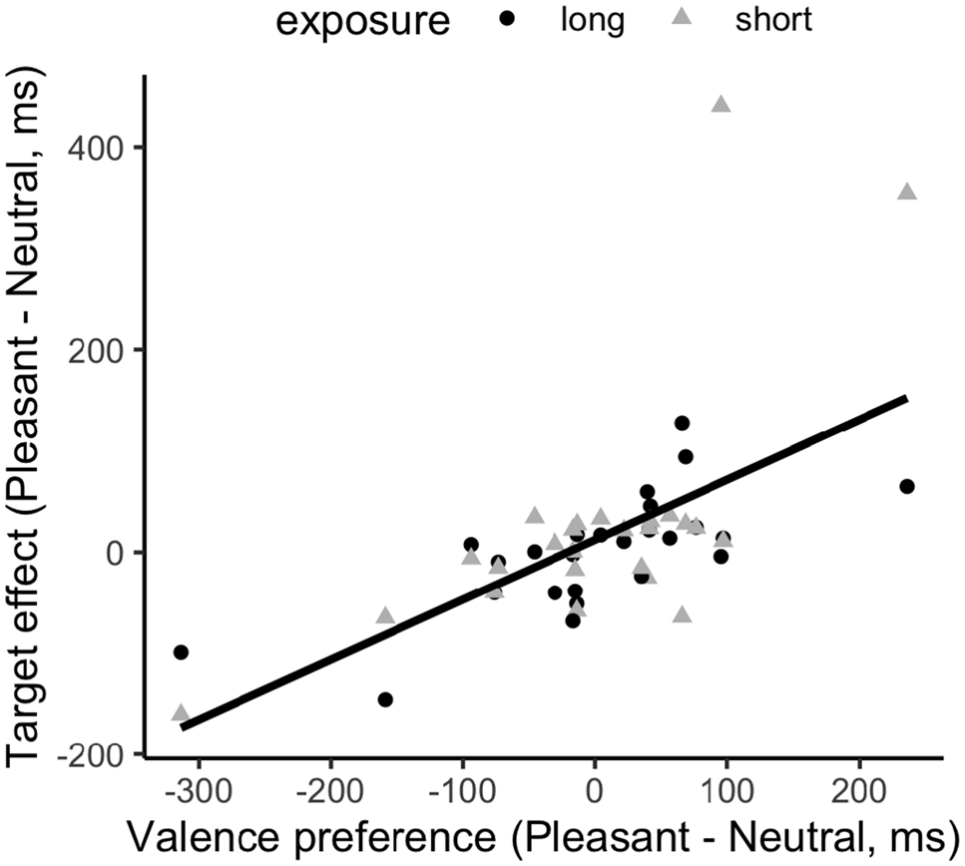
Correlation between the color–valence preference in the association phase and the target (facilitation) effect in the test phase, across the long- (circle) and short-exposure (triangle) conditions

The reliability analysis revealed the valence-preference score during the association session to be high, *r* = 0.71 with 95% CI [0.47, 0.85], and medium to high during the test session, with 0.37, 95% CI [− 0.03, 0.63] for the long-exposure session and 0.57, 95% CI [0.21, 0.74] for the short-exposure session. Despite the lack of an effect of the valence association in the averaged RTs, there was again a strong correlation between the color–valence preference in the association phase and the valence-based facilitation in the test phase (*r* = 0.63, *p* < 0.001), with a slope of 0.59; see Fig. 8. Corroborating the findings of Experiments 1 and 2, this correlation is indicative of a stable (pre-established) color–valence preference that carries through from the association to the test phase of the experiment. Note, however, that the strength of the linear relationship was greater in Experiment 3 than in Experiments 1 and 2 (slopes of 0.59 vs. 0.119).

### Discussion

Experiment 3 set out to test whether any color–emotion linkage established in the association phase would facilitate performance in the test phase if the happy- or, respectively, neutral-face-associated color appears (as an irrelevant feature) in the shape-defined target. In the association phase, red targets were responded to significantly faster than green targets; but there was no significant modulation of RTs by the differential (pleasant vs. neutral) valence associated with the two colors; this pattern is consistent with Experiments 1 and 2, as well as earlier reports (Anderson, 2016, 2017; Kim & Anderson, 2020). In the test phase, RTs to the shape-defined (diamond) target were greatly expedited if it possessed one of the colors that had previously been associated with happy- or neutral-face feedback, compared to a non-associated color. This shows that a previously (in the association phase) critical and emotionally rewarded target feature boosts search performance even when this feature is rendered non-critical for performing the task, in line with a general selection-history effect (Sha & Jiang, 2016); that is, the facilitation of shape-search by the two emotional-face-associated (as compared to non-associated) colors likely arises from a persistent bias in the system in favor of the previously relevant colors (irrespective of their emotional-valence association), which boosts the priority of the shape (saliency) signal (cf. Krummenacher et al., 2001, 2002).

Critically, however, the mean facilitation effect was not modulated by the emotional valence paired with the accidental target color in the association phase (i.e., it was not greater for happy- vs. neutral-face-associated colors). However, a correlation analysis between the color–valence preference in the association phase and facilitation in the test phase revealed a significant positive relationship, consistent with the findings of Experiments 1 and 2: those who showed an RT advantage (or, respectively, disadvantage) for the happy- relative to neutral-face-associated color in the association phase also showed an advantage (or, respectively, disadvantage) in the test phase. This again is as predicted if the congruency between the color–valence pairing and individuals’ preference is the major factor determining the effect pattern in the test phase.

### Omnibus analysis

The sample sizes of the above three experiments were calculated based on previous studies that reported a main effect of the valence-based manipulation. Given the inconsistent findings in the extant literature, the valence-based association and attentional-interference effects may be too small to be picked up by the sample size we originally calculated. To check whether there would be any consistent results across experiments, we pooled Experiments 1, 2, and 3 together, which yielded a sample size of 99.

**Fig. 9:**
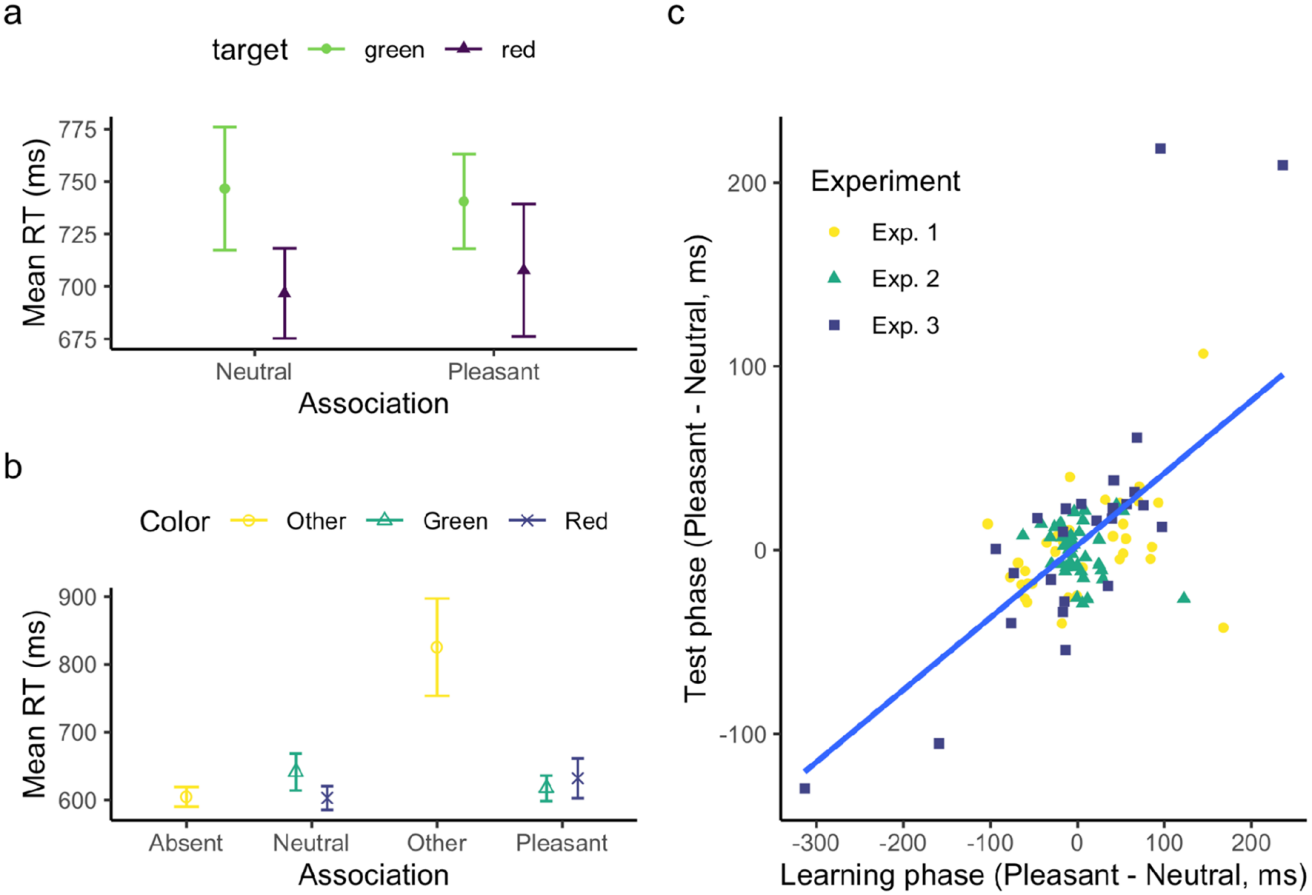
**a** Mean RTs, and associated SEs, for the association phase, collapsed across the three experiments, separately for the target color and valence association. **b** Mean RTs, and associated SEs, for the test phase, collapsed across the three experiments, separately for the distractor (Exps. 1 and 2)/target (Exp. 3) color–valence association. ‘Absent’ denotes the distractor-absent condition in Exps. 1 and 2, ‘Other’ the non-valence-associated color of the target in Exp. 3. **c** Correlation between the color–valence preference in the association phase and the distractor (interference; Experiments 1 and 2)/target (facilitation; Experiment 3) effect in the test phase, with the color of the individual participants’ data points corresponding to the three experiments. The color–valence preference was calculated as the RT difference between the pleasant- and neutral-emotion-associated target conditions in the association phase, and the (facilitation) effect as the difference between the pleasant and neutral-emotion-associated distractor/target conditions in the test phase

Figure 9a depicts the average RTs as a function of the target color and the associated feedback valence in the *association* phase, showing consistent facilitation of RTs by the red, but not the green, target color. A mixed-model ANOVA with the factors of Target Color and Color-Valence Association revealed only the Target-Color main effect to be significant, *F*(1, 171) = 19.92, *p* < 0.001, ${\eta }_{p}^{2}$ = 0.10, *BF* = 313.1. The main effect of Valence Association and the Color × Valence-Association interaction were non-significant, *F*s < 1.03, *p* > 0.31, ${\eta }_{p}^{2}$ < 0.01, *BF* = 0.032.

Figure 9b depicts the average RTs by the Color and Valence Association in the *test* phase. Given that the (color) distractor-absent condition in Experiments 1 and 2 differed from the ‘Other’ (color) condition in Experiment 3, we restricted our analysis to the Distractor/Target Color and Valence Association deriving from the association phase. A mixed-model ANOVA of the relative RTs, with the factors Color and Valence Association, revealed neither main effects to be significant: Color, *F*(1, 171.39) = 3.7, *p* = 0.056, ${\eta }_{p}^{2}$ = 0.02, *BF* = 0.09; Valence-Association, *F*(1, 171.39) = 0.188, *p* = 0.66, ${\eta }_{p}^{2}$ < 0.01, *BF* = 0.01. Nonetheless, the Color × Valence-Association interaction was significant, *F*(1, 179.05) = 8.98, *p* = 0.003, ${\eta }_{p}^{2}$ = 0.05, *BF* = 2.79, showing opposite RT patterns for the red and green colors (Fig. 9b); the underlying cause, however, remains unclear. Importantly, combining all experiments together did not reveal any significant valence-based attentional capture or facilitation. In fact, Bayes-factor analysis favored the null hypothesis.

Figure 8c depicts the overall positive relationship between the color–valence preference in the association phase and the distractor (interference; Experiments 1 and 2)/target (facilitation; Experiment 3) effect in the test phase. The correlation analysis revealed a significant positive correlation,* r* = 0.62, *p* < 0.001.

Thus, the omnibus analysis revealed a consistent color effect and a positive correlation between the color–valence preference in the association phase and the distractor-interference/target-facilitation in the test phase, while any effects of the experimental color–valence association remained undetectable (in both the association and the test phases) even with the increase of the sample size to 99. Nevertheless, it is crucial to approach these findings with caution, as the significant correlation was primarily driven by Experiments 1 and 3.

### General discussion

The present study aimed to reassess the influence of learnt color–emotion associations in a value-driven attentional-capture (visual-search) paradigm. Pleasant and neutral faces (in Experiments 1 and 3) and social-evocative images (in Experiment 2) served as feedback stimuli during the initial association phase. A growing body of work employing variants of this paradigm has shown that previously neutral stimuli, such as arbitrary target colors, when consistently paired with high-value (like monetary rewards) during the initial association phase, can subsequently interfere with performance in a test phase where color is task-irrelevant. This interference is attributed to robust attentional capture (Anderson et al., 2011; Le Pelley et al., 2015; Raymond & O’Brien, 2009; Sussman et al., 2013; Watson et al., 2019). Yet, there has been no reliable evidence of valence-modulated interference effects in *emotion*-associated attentional-capture paradigms where social–emotional (predominantly face) pictures served as reward stimuli during the association phase. In fact, demonstrating significant valence-*dependent* attentional-capture effects has proven to be challenging (e.g., Anderson, 2017; Kim & Anderson, 2020). Simple and clear manipulations of monetary reward (high vs. low), typically signaled by textual feedback (like “10 cents”), are universally comprehensible. However, emotional photographs (such as faces expressing happiness or neutrality) vary in numerous ways (gender, ethnicity, attractiveness, etc.). While these images can be collectively described using basic emotional valence and arousal dimensions, their variability can lead to considerable inter-individual differences in the color-to-emotional-reward associations formed during the association phase. This individual variability, in turn, impacts the valence-dependent attentional interference.

It is also worth mentioning that the potential emotional associations of colors themselves have been largely overlooked in the literature (Jonauskaite et al., 2019a, 2019b, 2020; Maier et al., 2009). These studies demonstrate potential participants’ preferences in the selection paradigm. In this regard, these preferences may influence the relationship between color and visual search. For example, prior studies exploring experimentally manipulated color–emotion associations (Anderson, 2016, 2017; Anderson & Halpern, 2017; Anderson & Kim, 2018; Anderson et al., 2011; Cho & Cho, 2021; Kim & Anderson, 2020; Qi et al., 2013; Sha & Jiang, 2016) primarily used *red* and *green* as the key colors. These colors, having likely strong pre-existing associations and subjective preferences, might impede the establishment of any arbitrary associations intended to be formed during the experimental association phase. For instance, for some people, associating ‘red’ with a positive or neutral emotion might be more challenging than ‘green’, and vice versa for others. This could make acquired association effects difficult to demonstrate in the data averaged across the two colors, as is typically done in the literature.

Given this context, our experiments, while also using the same critical colors (red and green), aimed to examine valence-dependent modulations of attentional capture *separately* for the two colors in the average data. We sought to create experimental conditions that would either promote the acquisition of color–valence associations (such as the use of high-arousal feedback stimuli) or allow attentional-capture effects to be more evident (such as performing the task under time pressure during the test phase, which could hinder distractor control processes). In addition to examining average data, we conducted correlation analyses to determine individuals’ color–valence preferences in the association phase and valence-associated distractor interferences and target facilitations in the test phase.

Across all three experiments, we failed to find any significant influence of emotional feedback on performance during the association phase, even though the reliability measure was high across experiments. Furthermore, we did not observe a valence-dependent modification in distractor-interference (Experiments 1 and 2) or target-facilitation (Experiment 3) effects during the test phase, as per the average-based measures. This was the case despite implementing conditions intended to foster such effects, particularly: the introduction of time pressure in the test phase through short (as well as long) display exposure durations, and the inclusion of a high-arousal emotional-feedback condition in the association phase (in addition to a low-arousal one). Even though the RTs were faster with short test displays compared to the long ones, there was no interaction between this effect and Valence, and no interaction between this effect and Arousal. Therefore, we conclude that any valence-dependent performance adjustments, as assessed by the average-based measures, are likely to be quite weak (confirmed by the small Bayes-factor values in the omnibus analysis), if they exist at all. Nonetheless, there was a consistent color difference in the average-based measures. During the association phase, responses to red targets were consistently faster than those to green targets.

The comparable performance observed with pleasant and neutral-valence feedback during the association phase might appear surprising, as emotional stimuli are generally perceived as attention attractors (Bradley et al., 2012; Dominguez-Borràs & Vuilleumier, 2013; Hinojosa et al., 2015) and are often favored over neutral ones (Alpers, 2008; Calvo et al., 2007). Additionally, emotional stimuli (‘distractors’) have been found to influence the oculomotor system at an early stage, automatically and involuntarily directing eye movements (Bucker et al., 2015; Le Pelley et al., 2015; Nissens et al., 2017; Watson et al., 2019). However, the lack of a color–valence association effect (i.e., faster RTs to targets associated with pleasant vs. neutral valence) during the association phase is not uncommon. The fact that these instances seem more frequent in studies using emotion feedback (Anderson, 2017; Anderson & Halpern, 2017; Cho & Cho, 2021; Kim & Anderson, 2020), rather than monetary feedback, may be attributable to considerable differences in individual reactions to emotional feedback. Alternatively, the emotional-reward stimuli presented after the search task might themselves be so captivating or attention-grabbing that they are not consistently associated with the color of the search target during the association phase. Perhaps, even more critical is the likelihood of observers having relatively stable valence preferences for one color over another, developed over their lifetimes, which they bring along to the experiment. These pre-established preferences might be too ingrained to be influenced by the association of colors with pleasant or neutral emotional feedback in the association phase. In contrast, monetary-reward manipulations enable more effective *ad-hoc* associative learning as, for most observers, there are no pre-established associations of certain colors with high or low monetary rewards. For these reasons, it is unsurprising that we did not find evidence of differential distractor interference or target facilitation between the pleasant and neutral-valence conditions during the test phase, when considering average measures.

Interestingly, we observed across experiments a positive correlation between color–valence preference in the association phase and the valence-related effect in the test phase, despite no differential effects in the average measures. As mentioned earlier, the considerable variability evident in participants’ color–valence preferences, which show a nearly equal distribution from ‘negative’ to ‘positive’ biases (see Fig. 9c), suggests that the emotional feedback manipulation was relatively weak as compared to the color–valence preference. Participants without a strong preference would have been influenced by the experimentally manipulated color–valence pairing in one direction or the other. However, the data points are almost equally spread for participants near the zero preference point. The positive correlation was primarily driven by those participants who revealed a pronounced color–valence preference in the association phase, either in the positive or negative direction.

It is crucial to highlight that the positive correlation reflects a response-*facilitation* effect based on the congruity of the color–valence preference with the emotional-feedback manipulation, rather than an *interference* or attentional-capture effect seen with monetary-reward manipulations (Anderson et al., 2011). Yet, it remains unclear which processing stage(s) between attentional selection and response execution is facilitated by the presence of the valence-preferred color, even in a distractor. It is quite plausible that the presence of such a valence-preferred color is registered pre-attentively and enhances performance through activation of the feature-unspecific alerting system (e.g., Matthias et al., 2010; Weinbach & Henik, 2014), without causing unintentional attentional capture if the color appears in a distractor rather than the target. This notion aligns with Treisman’s ‘feature integration theory’ (cf. Treisman & Gelade, 1980), which posits that detection can occur without localization. In other words, the presence of a unique feature may be registered by a spatially parallel mechanism (pooling activity across a particular feature map, e.g., the map for ‘red’) without initiating an attentional orienting response that would localize the item. In Experiments 1 and 2, where the critical color always appeared in a distractor (unlike in Experiment 3, where it was in the target), a proactive color-based filtering mechanism may have blocked orienting to color signals (Allenmark et al., 2019; cf. Liesefeld & Müller, 2021; Müller et al., 2009; Won et al., 2019, 2020; Zhang et al., 2019, 2022). As such, even if the critical color did not generate a priority signal that summoned attention to its location (‘attentional capture’), it may have been detected pre-attentively, thereby activating the emotional (color–valence) representation system, alerting the system to the presence of an emotionally/motivationally significant item. This could have subsequently enhanced the later focal-attentional processing stages, including analysis of the target (which, as a singleton item, does produce a strong priority signal and gets selected) for the response-relevant feature and selecting and executing the appropriate motor response.

To justify the assumption of color-based filtering during the test phase, consider that the heterogeneity of item colors in the test display could lead to ‘spurious’ local color-feature contrast, likely occurring at one or more of the non-target locations (e.g., Liesefeld & Muller, 2021).^4^ These signals, once transferred to the priority map, could compete with the shape-driven target signal. To ensure efficient target selection in this context, potentially distracting ‘noise’ signals from the color dimension may be generally suppressed or ‘down-weighted’. As a result, priority computation would be largely or entirely driven by the shape contrast engendered by the target singleton (Liesefeld & Müller, 2021; Müller et al., 2009; Won et al., 2019, 2020; Zhang et al., 2022). Operating a strategy of color-signal suppression would be particularly beneficial to target detection if valence-preferred colors stand out more than other colors due to long-term color–valence learning. For instance, the feature-coding system may have become more sensitive to valence-preferred colors, either due to lower level perceptual learning or long-term memory-based biasing of the relevant feature detectors. Therefore, down-scaling of color signals in the priority computation would curtail the potential of items appearing in the valence-preferred color (‘distractor’) to *capture attention,* because the competition for selection is almost always resolved in favor of the unique shape signal (Allenmark et al., 2019; Qiu et al., 2023; Tsai et al., 2023), making it hard to demonstrate attentional-interference effects.

However, the potential benefits of down-weighting color signals become less clear when a potentially valence-preferred color appears in the target item, as in the case of Experiment 3. In this situation, having a valence-preferred color in the target could provide a ‘redundant’ signal adding to supplement the critical target (shape) signal in the computation of search priority (a ‘supra-dimensional’ signal; e.g., Itti & Koch, 2000; Itti et al., 1998; Liesefeld & Müller, 2020; Wolfe, 2021)—accelerating attention allocation to the response-critical item (e.g., Krummenacher & Müller, 2014; Krummenacher et al., 2001, 2002; Nasemann et al., 2023). Assuming that color signals are not down-weighted under these conditions, we would expect a differential facilitation effect by the valence-preferred (vs. the non-preferred) color in Experiment 3. The results indeed confirmed such an effect pattern, showing the highest positive correlation and the steepest regression slope out of all three experiments.

However, it is also possible that the strong correlation observed in Experiment 3 was also driven by the search history, as the valence-associated colors were task-relevant in both the association and test phases. In fact, substantial facilitation was observed even when the target appeared in the *non-preferred* color as compared to the *non-associated* color. This implies that the majority of the facilitation is attributable to a classical search-history effect—the fact that the two alternative (i.e., the preferred or non-preferred) colors were target-defining during the association phase (Sha & Jiang, 2016). In other words, owing to their historical status as targets, detecting the ‘old’, red- or green-colored targets in the test phase was expedited relative to detecting the other, ‘new’ colored targets.

Still, a search-history account would need to assume that color signals were suppressed in Experiments 1 and 2 (where no evidence consistent with a search-history effect was found), but not in Experiment 3. Such an account would also need to clarify why, in Experiment 3, targets in the preferred color produced greater facilitation than those in the non-preferred color. This latter effect might be attributable to the preferred color being perceptually more salient (as a result of long-term color–valence learning), thereby adding a stronger color signal to the crucial target-*shape* signal and increasing the target’s selection priority. Alternatively, the color might provide a non-specific boost to target processing at multiple stages, through the same mechanism discussed earlier in relation to the ‘color-distractor’ Experiments 1 and 2. Further research, such as analyzing event-related-potential (ERP) components thought to indicate pre-attentive target-selection and post-selective response-decision processes, would be required to distinguish between these alternative accounts.

There also appear to be a few guidelines that emerge from the present study for future work exploring emotional-valence-modulated attentional-capture effects. In particular, future work should employ colors (other than red and green) for which observers have no strong pre-established preferences (the particular colors may have to be individualized for particular observers, rather than using the same colors for all observers). The use of such colors may allow the effects of short-term valence-based manipulations to become demonstrable. Alternatively, one may focus on participants who do not strongly prefer one or the other pre-selected (and across observers) fixed colors. Whichever approach is chosen, there appears a need for establishing observers’ color–valence preferences before (or in any case: independently of) the experimental association phase (e.g., using appropriate psychometric tests), perhaps assessing both stable ‘trait’ measures as well as momentary ‘state’ fluctuations, both of which may impact the effects of the experimental color–valence manipulation. Furthermore, the moderate-to-low reliability observed in the distractor-interference measures in (the test phase of) Experiments 1 and 2 warrants cautious consideration of conclusions based on average valence-based interference.

## Conclusion

In summary, our findings suggest that the traditional averaging measures may not accurately reflect the relationship between the valence-based association and attentional-interference/facilitation effects. Instead, as revealed by our alternative correlational approach, there is a consistent positive correlation between the valence preference measured in the association phase and the valence-dependent performance in the test phase across all three experiments. This indicates that participants’ preference for certain color–valence associations predominantly acts in a facilitatory fashion, enhancing (rather than hampering) search performance when the preferred color is present anywhere in the display (even in a distractor). We take this to suggest that the facilitation effect is mediated through the general alerting system, boosting the chain of processes involved in the attentional selection and post-selective processing of the target item. Individuals’ color–valence preference seems to reflect long-term, stable color–valence associations, which are not significantly modulated by short-term experimental pairings of colors with differently valenced emotional stimuli.

## Data Availability

All data and analysis scripts are available at: https://github.com/msenselab/valence_color_visual_search.

## Footnotes

1 The colors were adjusted to be of near-equal luminance on the local laptop used to program the experimental code. Nevertheless, given that Experiment 1 was run online, equiluminant stimulus presentation could not be ensured on the participants’ individual computers. However, in the lab-based Experiments 2 and 3, we took care to minimize any confounding luminance differences potentially contributing to physical item saliency (see Method of Experiment 2).

2 The ‘ignore-the-color’ instruction is typical in this type of study. Therefore, we modeled our instruction after Anderson et al. (2011), who told their participants that “color was irrelevant to the test phase and should be ignored” (p. 10368).

3 Due to one erroneous assignment, color–valence associations were slightly different. Specifically, in the high-arousal session, for 20 participants, a red color was paired with a neutral one, and a green color with a pleasant one; the reverse was true for the remaining 18 participants. The target color association with neutral versus pleasant feedback was reversed across the high- and low-arousal sessions for the same participants.

4 An example would be an item array (section) *v-w-x-y-z* in which the central (colored) item ***x*** happens to be flanked by items of a relatively distant color on the color circle. If the flankers on each side (*v*, *w*, and *y*, *z*) are similar in color, item ***x*** would produce a relatively strong local (color-) feature-contrast signal (and the immediate flankers *w* and *y* a somewhat weaker signal), due to the operation of lateral iso-feature suppression (e.g., Li, 2002).
